# CropCircDB: a comprehensive circular RNA resource for crops in response to abiotic stress

**DOI:** 10.1093/database/baz053

**Published:** 2019-05-06

**Authors:** Kai Wang, Chong Wang, Baohuan Guo, Kun Song, Chuanhong Shi, Xin Jiang, Keyi Wang, Yacong Tan, Lequn Wang, Lin Wang, Jiangjiao Li, Ying Li, Yu Cai, Hongwei Zhao, Xiaoyong Sun

**Affiliations:** 1 Agricultural Big-Data Research Center, College of Information Science and Engineering, Shandong Agricultural University, Taian, China; 2 Department of Plant Pathology, Nanjing Agricultural University, Nanjing, China

## Abstract

Circular RNA (circRNAs) may mediate mRNA expression as miRNA sponge. Since the community has paid more attention on circRNAs, a lot of circRNA databases have been developed for plant. However, a comprehensive collection of circRNAs in crop response to abiotic stress is still lacking. In this work, we applied a big-data approach to take full advantage of large-scale sequencing data, and developed a rich circRNA resource: CropCircDB for maize and rice, later extending to incorporate more crop species. We also designed a metric: stress detections score, which is specifically for detecting circRNAs under stress condition. In summary, we systematically investigated 244 and 288 RNA-Seq samples for maize and rice, respectively, and found 38 785 circRNAs in maize, and 63 048 circRNAs in rice. This resource not only supports user-friendly JBrowser to visualize genome easily, but also provides elegant view of circRNA structures and dynamic profiles of circRNA expression in all samples. Together, this database will host all predicted and validated crop circRNAs response to abiotic stress.

## Introduction

Circular RNA (circRNA) was first reported to encode subviral agents in plants. In 2012, circRNAs were found to exist in human cells functioning as microRNA sponges and thus mediating expression of mRNA (1, 2). Later, circRNAs were further reported to exist widely in eukaryotes, including fungi, protists, plant, etc (3). Recently, a few teams have characterized circRNAs in plants, including *Arabidopsis* (4, 5, 6) and rice (7), and verified their important roles in alternative splicing (8). Although a lot of work has been done, the function of circRNAs remains unclear. Until now, it has been reported that circRNAs may mediate mRNA expression as miRNA sponges (1, 2), control the process of protein translation (9) or produce proteins directly via translation (10, 11).

Since the community has paid more attention to circRNAs, a lot of circRNA databases in human and animals have been developed. For example, Circ2Traits was developed to link circRNA with human disease and traits (12). Also, circBase collected thousands of circRNAs from nine independent studies, hosting circRNAs from human, mouse, nematode and latimeria. In addition, CircNet was the first circRNA database derived from large-scale sequencing data. Recently, TSCD, a tissue-specific circRNA database for human and mouse, was developed to host 302 853 tissue-specific circRNAs (13). Finally, CSCD, a cancer-specific circRNA database, was reported to contain 272 152 cancer-specific circRNAs, and 950 962 circRNAs in normal samples (14).

Simultaneously, three plant circRNA databases were reported to date. PlantcircBase collected publicly available 77 595 circRNAs, including rice, *Arabidopsis*, maize, tomato and barley (15). PlantCircNet hosted circRNAs originating from eight plant species, and offered plant circRNA-miRNA-gene regulatory networks (16). AtCircDB was developed by our group based on large-scale sequencing data in 2016 (27). This database hosted tissue-specific 30 648 circRNAs for *Arabidopsis* derived from 87 independent studies. However, to the best of our knowledge, a comprehensive and systematic collection of circRNAs for crops in response to abiotic stress is still lacking. Following our previous work (27), we applied a big data approach to take full advantage of large-scale sequencing data. We developed a rich stress-specific circRNA resource: CropCircDB (http://genome.sdau.edu.cn/crop/ or http://deepbiology.cn/crop/) for maize and rice, later extending to incorporate more crop species. This database currently hosts 38 785 circRNAs in maize, and 63 048 circRNAs in rice, which is freely available for download.

## Materials and methods

### Data collection

On 12 November 2017, we used different keywords, including ‘drought’, ‘cold’, ‘heat’, ‘salt’, ‘flood’ and ‘high wind’ to search for the RNA-Seq data sets stored in the NCBI SRA database (https://www.ncbi.nlm.nih.gov/sra) for two crops: maize and rice. Only three abiotic stresses (‘drought’, ‘cold’, ‘salt’) with >20 samples were kept for analysis. Later, we will extend to other abiotic stresses when more samples are publicly available. These samples are from diverse plant tissues, including root, leaf, flower, shoot, etc. In addition, we selected those sequencing data without ‘PolyA’ selection in the sample preparation. Finally, we only kept those samples with three criteria: they should (i) be sequenced with Illumina platform, (ii) have a file size >1 G and (iii) have identified circRNAs. The detailed information about the sequencing samples is available at the website.

In addition, we also searched PubMed (https://www.ncbi.nlm.nih.gov/pubmed) using ‘rice, circular RNA’ and ‘maize, circular RNA’. One maize (17) and three rice (7, 4, 18) articles provided detailed circRNA lists, which were collected and annotated. Also, this circRNA collection was made publicly available at our website.

### circRNA identification

To detect circRNAs, we utilized two algorithms: CIRCexplorer2 (19) and CIRI2 (20) with default parameters simultaneously to increase the prediction accuracy. In the CIRCexplorer2 pipeline, TopHat (21) was utilized to align the raw sequencing data to the reference genome with the following parameters: ‘–max-multihits 1 -a 6 --microexon-search -m 2’. Then, unmapped bam files were converted to fastq format using bam2fastx. TopHat was further used to process fastq files with the following parameters: ‘-p 15 --fusion-search --keep-fasta-order --bowtie1 --no-coverage-search’. Finally, the results were analyzed with CIRCexplorer2 with default parameter. In the CIRI2 pipeline, the sequencing data was first aligned to the reference genome with BWA-MEM with the following parameter: ‘-T 19’ (22). Then, CIRI2 was applied to alignment file (SAM format) to detect circRNAs. All circRNAs detected were then annotated using SplicingTypesAnno (23) and Bioconductor package: GenomicAlignments (24). In addition, we extracted all the circRNA sequences using BEDTools (25) and used Bioconductor package: Biostrings (26) to predict amino acid sequences from spliced sequences following (10) work.

### Detection score and stress detection score

Following our previous approach (27), we used ‘detection score’ to measure the existing robustness of a circRNA in the sample. To further understand the existence of circRNAs under abiotic stress, we designed a new metric: ‘stress detection score’ as follows.

$$ detection\ score$$$$\qquad=\frac{\mathrm{the}\ \mathrm{number}\ \mathrm{of}\ \mathrm{samples}\ \mathrm{containg}\ \mathrm{a}\ \mathrm{circRNA}}{\mathrm{total}\ \mathrm{number}\ \mathrm{of}\ \mathrm{samples}}\times 100 $$

$$ stress\ detection\ score$$$$\qquad=\frac{\mathrm{the}\ \mathrm{number}\ \mathrm{of}\ \mathrm{stressed}\ \mathrm{samples}\ \mathrm{containg}\ \mathrm{a}\ \mathrm{circRNA}}{\mathrm{total}\ \mathrm{number}\ \mathrm{of}\ \mathrm{stressed}\ \mathrm{samples}}$$$$\times 100 $$

If this score is 100, it means that this circRNA was detected in all the related samples; if this score is 0, it suggests that this circRNA was not found in all the related samples. This metric helps experimental biologist to rank the circRNAs and design further analysis.

### Analysis of miRNA-circRNA interaction

To understand the relationship between miRNA and circRNA, we downloaded the microRNA information from miRBase (http://mirbase.org/). Then, we extracted all the miRNA and circRNA sequences using Bioconductor package: Biostrings and BEDTools. To predict the interaction between miRNAs and circRNAs, we utilized psRNATarget (28) and uploaded the circRNA sequences to the website (https://plantgrn.noble.org/psRNATarget/analysis?function=2). After choosing the scoring schema with default parameters and analyzing the results, we extracted the potential interactions between miRNAs and circRNAs using R. The final results were annotated and deposited into the database.

### Super circRNA region

To help group the circRNAs, we followed our previous approach (27) and used super circRNA regions to cluster those circRNAs originating from the same genome locus. The pipeline is as follows: firstly, we collapsed all the overlapped circRNAs into one region, and defined the number of circRNAs in one region as *C_i_*. Secondly, we analyzed the *C_i_* using five-number summary (min, Q1, median, Q3, max). Then we calculated the super circRNA regions as those regions containing the number of circRNAs more than *C_Q1_* + 1.5(*C_Q3_* − *C_Q1_*). The final results were annotated and deposited into the database.

### Database construction

We used PHP and MySQL to develop the database. The genomic visualization was accomplished through JBrowser (29). Two tracks including gene and circRNA annotation were added. The gene track was imported using GFF3 files downloaded from EnsemblPlants (http://plants.ensembl.org/). The circRNA track was imported using annotation from the previous section: ‘circRNA identification’. In addition, circRNA structure and expression visualization was implemented using JavaScript (D3, https://d3js.org/). The circRNA structure was developed with exon annotation and generated dynamically with regards to each circRNA. The expression visualization was generated based on the circRNA expression (RPM: reads per million mapped reads) labeled with sample ID from NCBI SRA database. To improve the website usage, we not only developed a website (http://genome.sdau.edu.cn/crop/) hosted in the university, but also implemented a mirror site at the commercial organization (http://deepbiology.cn/crop/).

## Results and Discussion

**Figure 1:**
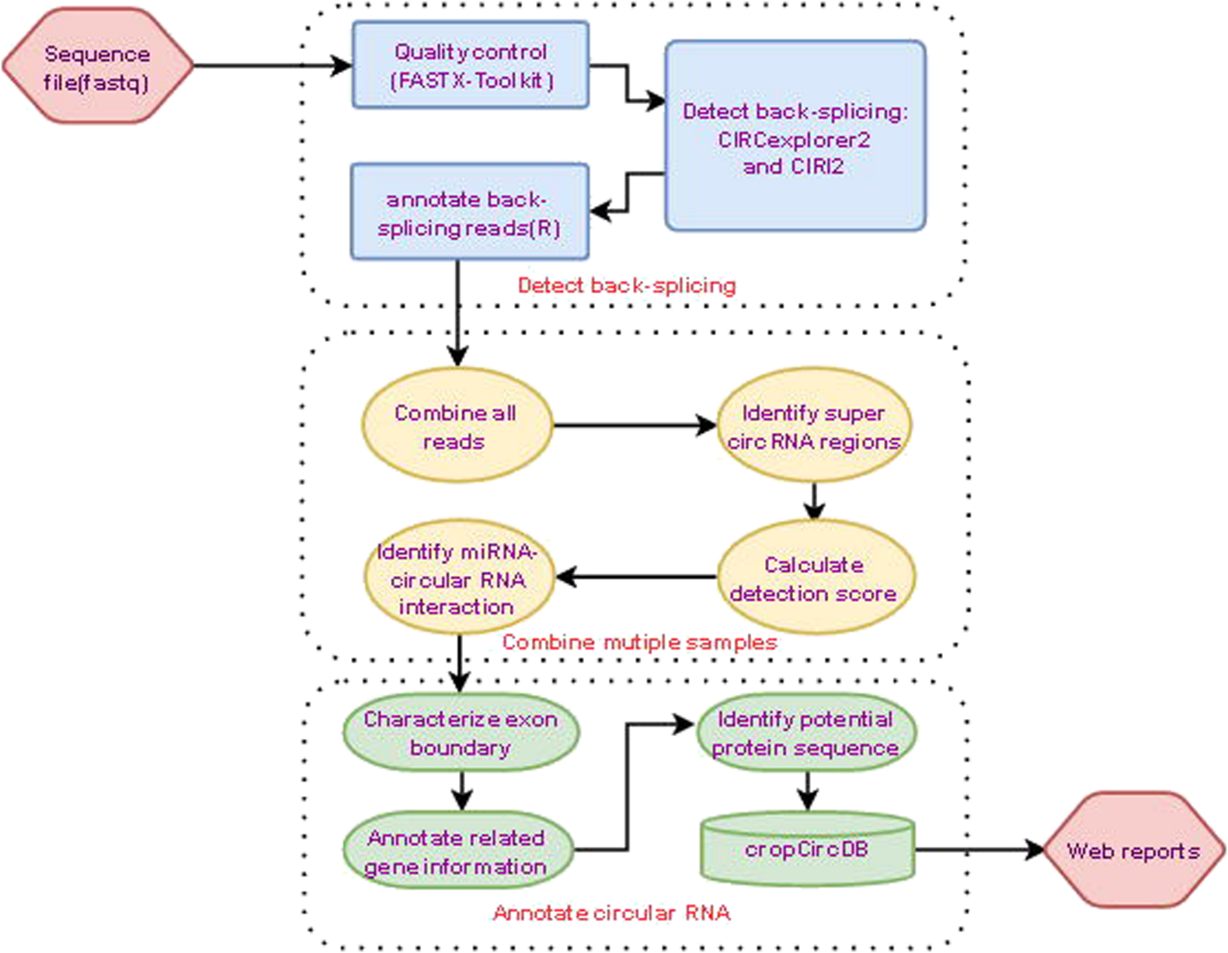
Analysis pipeline.

In this study, we systematically investigated 244 maize samples and 288 rice samples, including leaf, root, shoot, etc. All samples were downloaded from NCBI SRA database, and the circRNAs were detected with two algorithms: CIRCexplorer2 and CIRI2. The circRNAs were further processed by psRNATarget to predict potential miRNA target sites (Figure 1). In total, we found 38 785 circRNAs in maize, and 63 048 circRNAs in rice. The median length of circRNAs for maize and rice is around 261 nt and 260 nt, and the proportion of genes hosting circRNAs is 27% and 38%, respectively. Also, more than half of circRNAs originate from one exon, suggesting that circRNAs are generated from fewer exons, which is in line with our previous finding (27). Notably, 85% and 75% of circRNAs overlap with the exon boundaries while 4% and 3% of circRNAs originate from intergenic regions for maize and rice, respectively.

To investigate the environmental effects on circRNAs, we systematically analyzed 111 stress-related maize samples and 148 stress-related rice samples (Table 1). Specifically, for maize, we collected 85 drought samples versus 73 control samples, and 23 salt samples versus 4 control samples. Similarly, for rice, we collected 60 drought samples versus 47 control samples, 29 salt samples versus 29 control samples and 46 cold samples versus 16 controls. Together, we found 12 643 and 15 588 circRNAs in the control samples for maize and rice, respectively. In addition, for maize, we found 11 206 drought-specific circRNAs, and 6770 salt-specific circRNAs. For rice, we found 824 drought-specific circRNAs, 6313 salt-specific circRNAs and 5724 cold-specific circRNAs. All the stress-related circRNAs as well as tissue information were deposited in CropCircDB.

**Table 1:** circRNAs in maize and rice

**Organism**	**Stress**	**Sample no.**	**circRNA no.**	**Total circRNA no.**
**Maize**	**Drought**	**85**	**11 206**	**38 785**
	**Salt**	**23**	**6770**	
	**Other**	**59**	**20 809**	
**Rice**	**Drought**	**60**	**824**	**63 048**
	**Salt**	**29**	**6313**	
	**Cold**	**46**	**5724**	
	**Other**	**61**	**50 187**	

**Figure 2:**
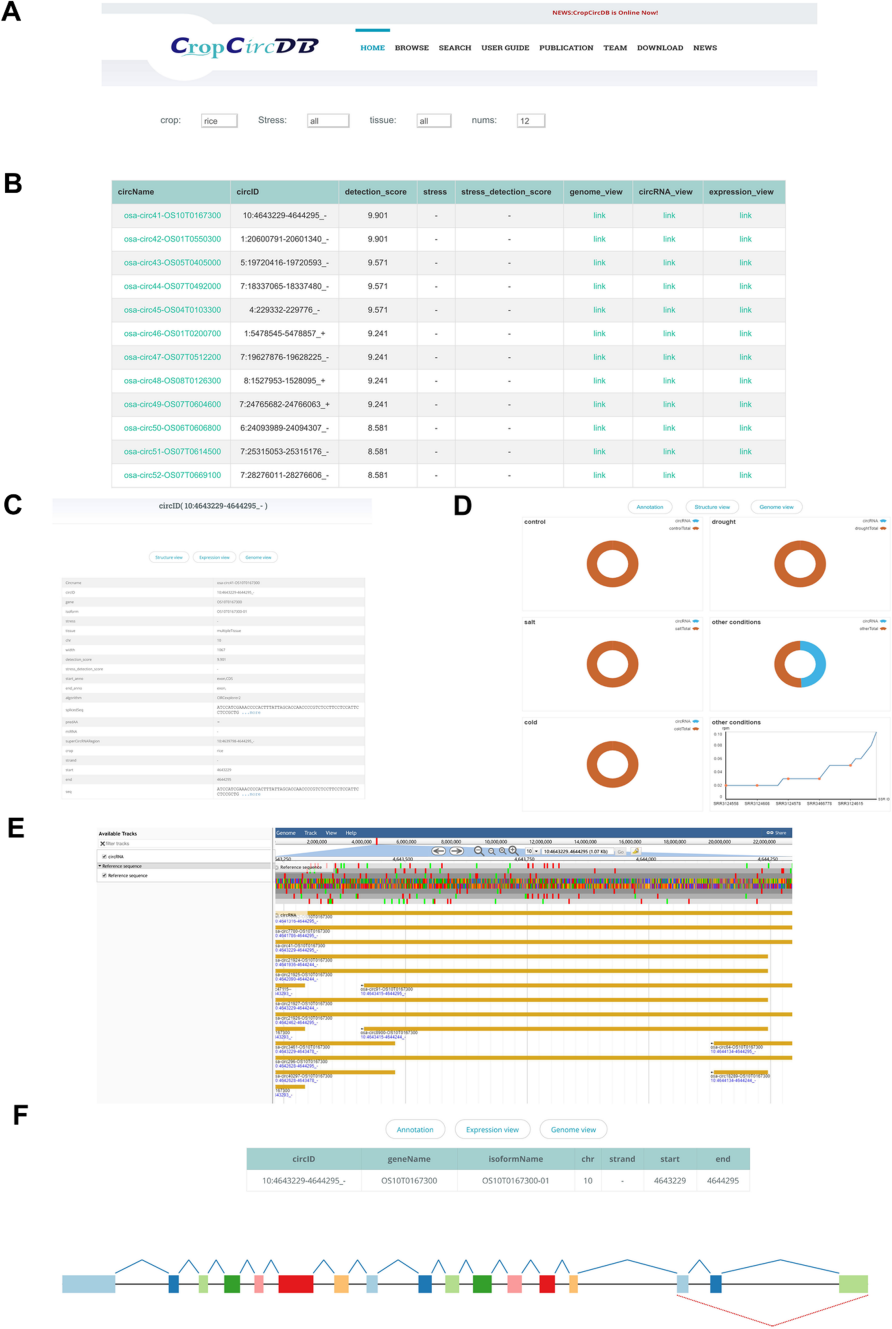
CropCricDB database.

**Figure 3:**
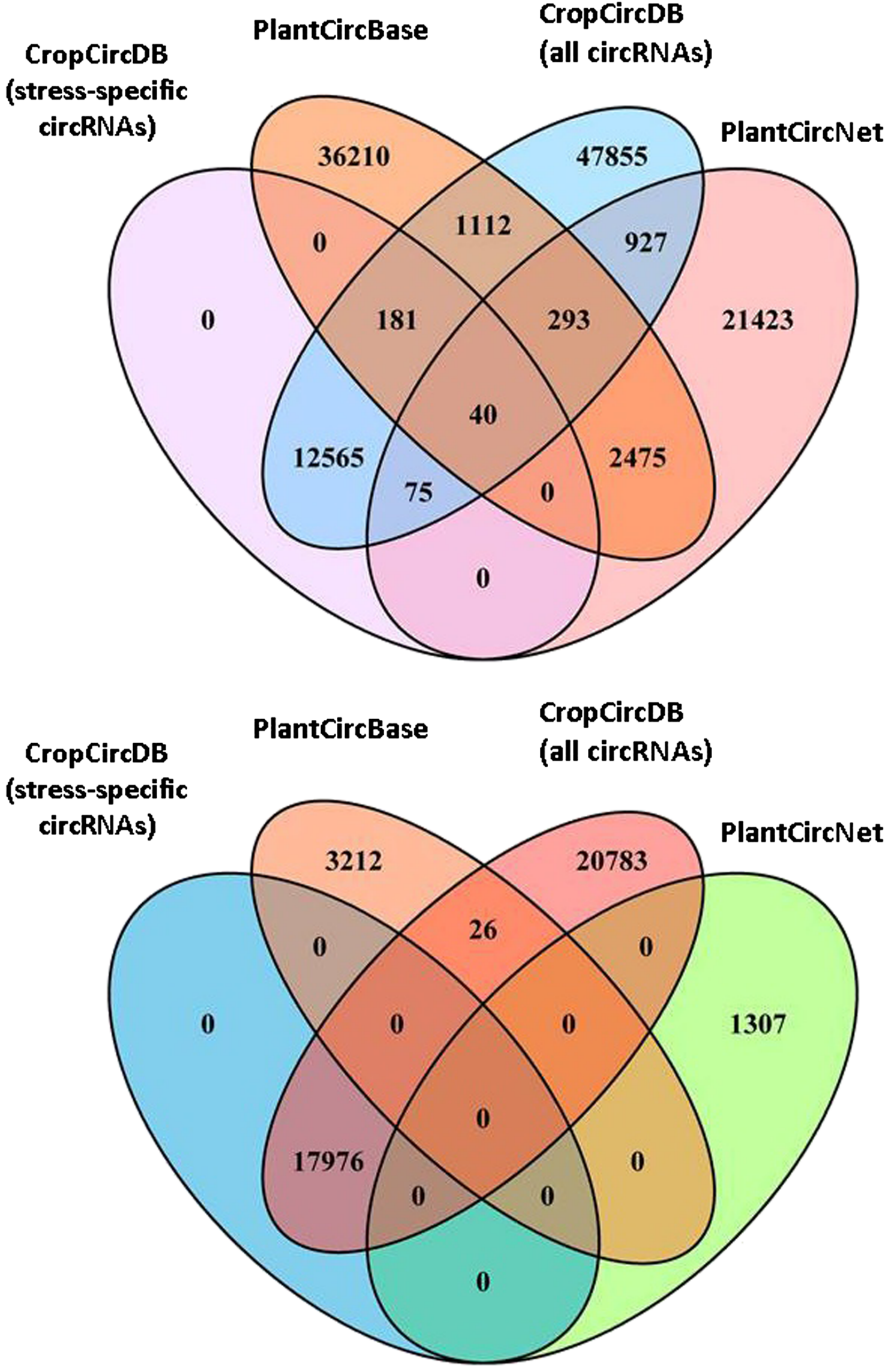
Comparison of three plant circRNA resources (CropCircDB, PlantCircBase, PlantCircNet). The top figure is for rice. The bottom figure is for maize.

To understand the relationship between circRNAs and protein, we extracted all the sequences of circRNAs using SplicingTypesAnno and bedtools. Next, we took off the intron sequences and kept all those spliced sequences. Following the approach of (11), we translated all RNA sequences into amino acid sequences. If there was no stop codon existing in the sequences, the amino acid sequences were stored as the predicted proteins (10). As a result, the database not only hosts the full sequences from the start to the end of circRNAs, but also the spliced sequences without introns as well as the predicted proteins.

Currently, CropCircDB provides the following information: (i) circRNA name. The naming system follows the previous work (30), and incorporates the species abbreviation, circRNA IDs and gene names together. This design not only links circRNAs with genes conveniently, but also allows users to compare, query, retrieve and store circRNA clusters; (ii) circRNA information, including chromosome, start, end, strand, length and antisense information; (iii) detection score and stress detection score. Detection score is a metric that we designed in a previous study (27) to measure the probability of circRNA found in the sample. This score is calculated by the number of samples with detected circRNA/total number of samples. A high detection score suggests that the probability of this circRNA is high in the sample. Similarly, to measure the robustness of circRNA found in the stressed samples, we developed the stress detection score, calculated by the number of stressed samples with detected circRNAs/total number of stressed samples; (iv) experimental evidence. All the validated circRNAs are annotated as ‘validated’, and the website also accepts the submission from the community. All new circRNAs will be deposited into the database in 24 hours; (v) potential interaction between circRNAs and miRNA. circRNAs are reported to function as miRNA sponges. We analyzed all the circRNAs using psRNATarget to search for potential interaction between circRNAs and miRNA. In total, we found 96 miRNAs interacting with 327 circRNAs for maize, and 518 miRNAs interacting with 5475 circRNAs for rice; (vi) super circRNA regions. These regions contain highly enriched circRNAs described in our previous study (27). To help users investigate these specific structures, we followed the same approach and extracted them with all the related circRNAs. As a result, we finally got 3030 and 5813 super circRNA regions for maize and rice, respectively.

The web interface of CropCircDB includes tutorial, browser, search, download, publication, team and news information (Figure 2A). The search portal is the main function of the website (Figure 2B and C). It currently supports three main features: (i) genome visualization (Figure 2E). JBrowser provides annotation for all the linear transcripts, including exon, transcripts and genes. To highlight the circRNAs, we also labeled the circRNAs from start to end, and users can compare circRNAs to other genomic features easily and conveniently by dragging in the panel; (ii) circRNA structure visualization (Figure 2F). By inserting the circRNA into the splicing schema, users can easily compare the circRNA structure with related exons from the same isoform; (iii) circRNA expression visualization (Figure 2D). The platform also supports pie charts, scatterplots and boxplots for visualizing the proportion of circRNAs found in control and stressed samples as well as the detailed expression value (RPM) in each sample.

Finally, we also compared our database to other two plant circRNA resources: PlantcircBase (15) and PlantCircNet (16). These three databases share a few thousands of circRNAs, and are complementary to each other (Figure 3).

## Conclusions

This database provides a comprehensive platform for circRNAs in maize and rice response to abiotic stress. It not only holds detailed information for circRNAs, including genome locus, gene name and isoform name, but also provides extended services, including detection score, super circRNA regions, miRNA interactions, experimental evidence and predicted proteins. Also, this platform supports user-friendly genome visualization through JBrowser and offers an elegant view for circRNA structure with regard to exons. Finally, it also provides dynamic profiles of circRNA expression in all samples. Users cannot only explore back splicing and canonical splicing in regards of exons and introns and examine whether or not the circRNAs are stress-specific, but also follow up this circRNA resource and unravel their functional and regulatory roles. We believe this resource will help the community gain deep understanding of circRNAs in crops.

## Author contributions

X.S. and H.Z. designed and supervised the project; X.S., K.W., K.S., C.S., K.W., R.L. and Y.T. analyzed the data; C.W., X.J., J.L. and Y.L. developed the database; X.S. wrote the manuscript.

## Supplementary Material

supplementaryData_baz053

## Notes

**Database URL:** http://deepbiology.cn/crop/
