# Supplementary material for: CropCircDB: a comprehensive circular RNA resource for crops in response to abiotic stress
Source: Database (Oxford). 2019 May 6;2019:baz053. doi: 10.1093/database/baz053 (PMC6501434; doi:10.1093/database/baz053)
Supplement: supplementaryData_baz053 [file supplementarydata_baz053.pptx]

## Slide 1
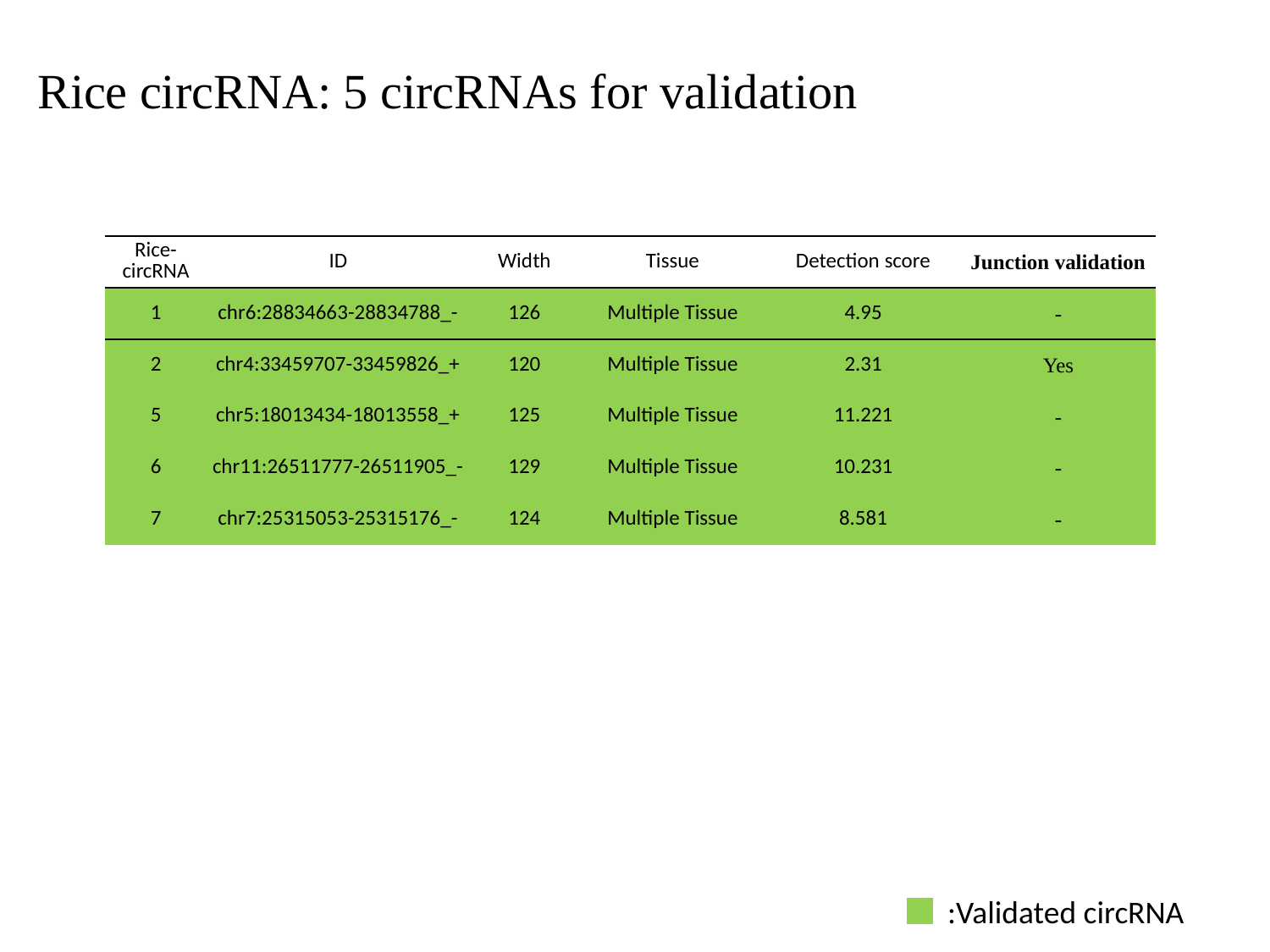

# Rice circRNA: 5 circRNAs for validation
| Rice-circRNA | ID | Width | Tissue | Detection score | Junction validation |
| --- | --- | --- | --- | --- | --- |
| 1 | chr6:28834663-28834788\_- | 126 | Multiple Tissue | 4.95 | - |
| 2 | chr4:33459707-33459826\_+ | 120 | Multiple Tissue | 2.31 | Yes |
| 5 | chr5:18013434-18013558\_+ | 125 | Multiple Tissue | 11.221 | - |
| 6 | chr11:26511777-26511905\_- | 129 | Multiple Tissue | 10.231 | - |
| 7 | chr7:25315053-25315176\_- | 124 | Multiple Tissue | 8.581 | - |
:Validated circRNA

## Slide 2
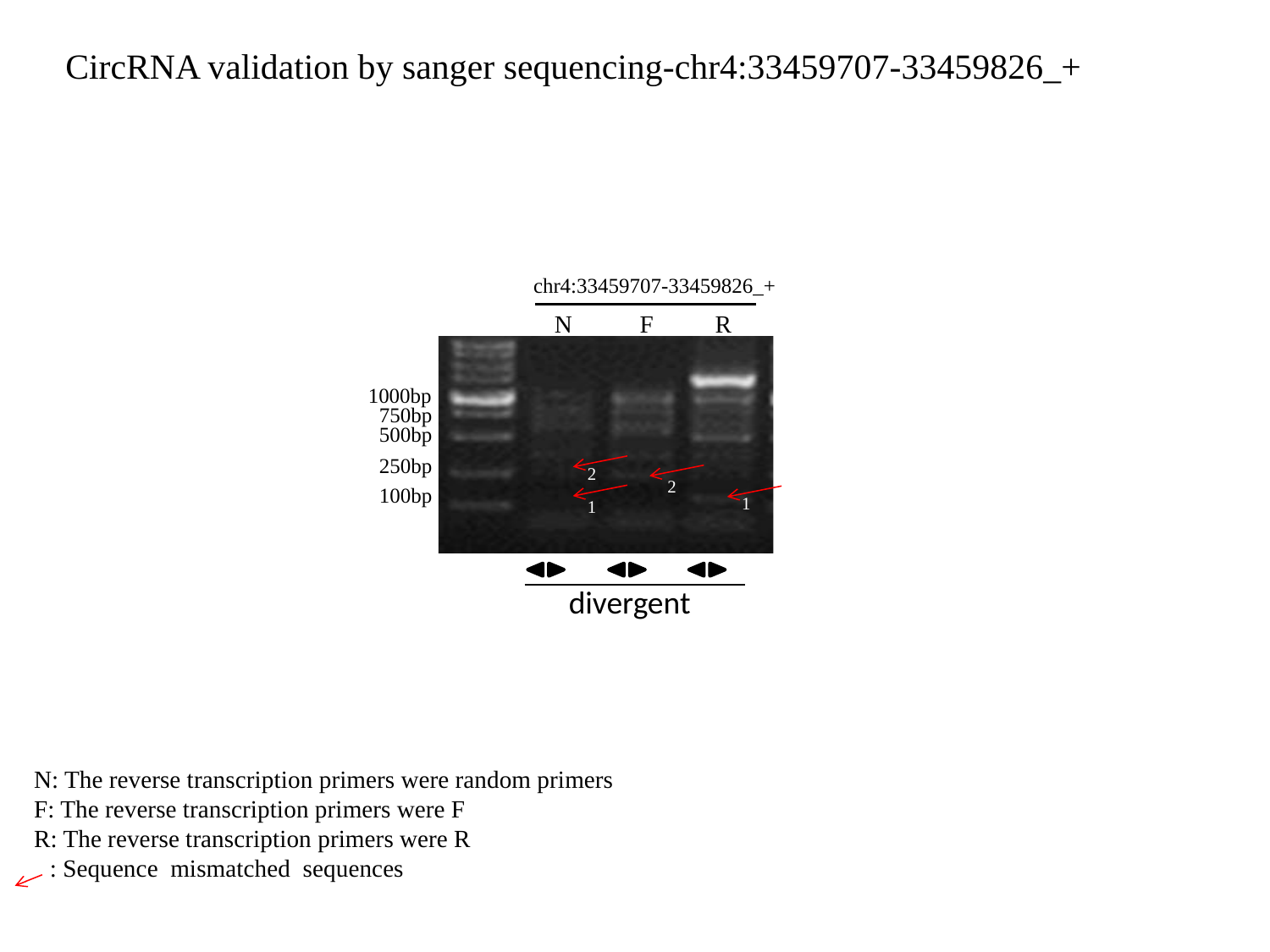

# CircRNA validation by sanger sequencing-chr4:33459707-33459826_+
chr4:33459707-33459826_+
 N F R
2
2
1
1
1000bp
750bp
500bp
250bp
100bp
divergent
N: The reverse transcription primers were random primers
F: The reverse transcription primers were F
R: The reverse transcription primers were R
 : Sequence mismatched sequences
